# Elevated Plasma Fibrinogen Is Associated With Excessive Inflammation and Disease Severity in COVID-19 Patients

**DOI:** 10.3389/fcimb.2021.734005

**Published:** 2021-08-03

**Authors:** Jingrui Sui, Denis F. Noubouossie, Sheetal Gandotra, Liyun Cao

**Affiliations:** ^1^Division of Laboratory Medicine, Department of Pathology, University of Alabama at Birmingham, Birmingham, AL, United States; ^2^Department of Hematology, the Affiliated Yantai Yuhuangding Hospital of Qingdao University, Yantai, China; ^3^Division of Pulmonary, Allergy, and Critical Care Medicine, Department of Medicine, University of Alabama at Birmingham, Birmingham, AL, United States

**Keywords:** fibrinogen, coronavirus disease 2019 (COVID-19), inflammation, cardiovascular disease, coagulation

## Abstract

**Background:**

The coronavirus disease-19 (COVID-19) is characterized with intense inflammatory response, cardiac involvement, and coagulopathy. Fibrinogen, as a biomarker for inflammation, cardiovascular disease, and coagulation, has not been fully investigated yet. The aim of this study was to assess the clinical application of fibrinogen in COVID-19 patients.

**Methods:**

We retrospectively analyzed the demographic and laboratory characteristics of 119 COVID-19 patients in the University of Alabama of Birmingham Medical Center. Correlations of fibrinogen on admission with intensive care unit (ICU) admission, disease severity, and laboratory parameters were analyzed.

**Results:**

Among the 119 COVID-19 patients, 77.3% (92/119) had severe disease, and 59.5% (71/119) patients were admitted to the ICU. Elevated fibrinogen was detected in 67.2% (80/119) of the patients. Fibrinogen levels were significantly associated with inflammatory markers and disease severity, but not with cardiac injury biomarker high sensitivity troponin I. Patients with severe disease had increased fibrinogen levels upon admission compared to patients with non-severe disease (*P* = 0.001). Fibrinogen level at 528.0 mg/dl was the optimal cutoff to predict disease severity, with a sensitivity and specificity of 66.7% and 70.3% (area undty -60er the curve [AUC] 0.72, *P* = 0.0006).

**Conclusions:**

Fibrinogen is commonly elevated in COVID-19 patients, especially in those with severe disease. Elevated fibrinogen correlates with excessive inflammation, disease severity, and ICU admission in COVID-19 patients.

## Introduction

Coronavirus disease-19 (COVID-19) caused by 2019-nCoV/SARS-CoV-2 has led to a global pandemic (Zhou et al., 2020b). While respiratory illness is the major cause of morbidity and mortality in the COVID-19 patients, coagulopathy (Wang et al., 2020; Zhou et al., 2020a), systemic inflammation (Inciardi et al., 2020) and myocardial injury (Aghagoli et al., 2020) are also involved in the pathogenesis of the disease. Elevated prothrombin (PT), activated partial thromboplastin time (aPTT) and D-dimer have been reported in COVID-19 patients (Chen et al., 2020b; Wang et al., 2020; Zhou et al., 2020a). During the disease progression, activation of the host defense system results in an imbalance between the procoagulant and anticoagulant systems (Liu et al., 2021). This leads to thrombi formation that is termed as immunothrombosis (Key et al., 1990; Engelmann and Massberg, 2013). D-dimer, as a fibrin degradation product, has been widely reported to be elevated in COVID-19 patients (Rostami and Mansouritorghabeh, 2020), though without standard reporting (Favaloro and Thachil, 2020; Thachil et al., 2020). However, fibrinogen as a clotting factor in coagulation and thrombosis, has not been well studied. In addition, fibrinogen acts as a positive acute phase reactants, which is increased during inflammatory response (Engelmann and Massberg, 2013). Furthermore, fibrinogen has been considered as an independent risk factor for cardiovascular disease (Ernst, 1990; Heinrich and Assmann, 1995; Sweetnam et al., 1996; Ahmed et al., 2012). It has been reported that fibrinogen levels are increased in COVID-19 patients (Arachchillage and Laffan, 2020). Additionally, autopsy studies of COVID-19 patients demonstrated the fibrin rich thrombi in small vessels and pulmonary capillaries (Fox et al., 2020). However, the overall profile of fibrinogen in COVID-19 patients has remained unclear. By analyzing the correlation of fibrinogen with disease severity, and laboratory markers, the goal of this study was to investigate the role of fibrinogen in the pathogenesis of COVID-19 and evaluate the role of fibrinogen as a biomarker for disease severity.

## Methods

### Study Participants and Data Collection

This study included patients with confirmed COVID-19 admitted to the University of Alabama at Birmingham (UAB) Medical Center (Alabama, United States) from March 19, 2020 to June 2, 2020 with fibrinogen tested on initial presentation. The institutional review board at UAB approved this study. The diagnosis was based on World Health Organization guidance and confirmed by 2019-nCoV/SARS-CoV-2 real-time reverse transcription polymerase chain reaction (RT-PCR) assay of nasopharyngeal or oropharyngeal swab samples in the clinical lab of UAB Medical Center (WHO, 2020, May 27). Patients’ demographic, clinical and laboratory data were retrospectively collected from the electronic health records. Clinically, severity of the COVID-19 patients was classified into mild, moderate, severe and critical disease according to the Clinical management of COVID-19 by World Health Organization (WHO, 2020, May 27). Patients were divided into non-severe (mild or moderate) and severe (severe or critical) groups. Severe disease was defined with clinical signs of pneumonia plus one of the following: respiratory rate >30 breaths/min, severe respiratory distress, SpO2 < 90% on room air. Laboratory parameters including complete blood count (CBC) with differentiation, coagulation profile, liver function, renal function, cardiac markers and inflammatory markers were obtained at initial diagnosis. Fibrinogen level was tested using immunoturbidimetric assay with a reference range of 220.0-498.0 mg/dl. Demographic information, clinical characteristics, laboratory parameters and outcomes were compared between patients with elevated and normal fibrinogen to evaluate the role of fibrinogen in COVID-19 patients.

### Statistical Analysis

All statistical analyses were performed with SPSS 26 and GraphPad Prism 8. Normally distributed continuous data were expressed as mean ± standard deviation (SD) and Student’s *t*-test was performed for data analysis. For data that were not normally distributed, values were expressed as median and interquartile range (IQR) and Mann-Whitney *U* test was performed to determine the differences between two groups. Categorical variables were expressed as number and percentage and compared by Fisher’s exact test. To assess the predictive value of fibrinogen for disease severity and ICU admission, receiver operating characteristic (ROC) analysis was conducted with the area under the ROC curve (AUC), sensitivity and specificity. The *P* values of < 0.05 and < 0.01 were considered to be statistically significant and highly significant, respectively.

## Results

### Baseline Clinical Characteristics and Laboratory Findings of the Study Population

During the study period, there were 552 laboratory-confirmed COVID-19 patients in UAB medical center. Among these cases, 119 patients with fibrinogen results on admission were enrolled in our study. The demographic, clinical characteristics, symptoms, and vital sign of the patients are shown in **Table 1**. The median age of patients was 62 years old (IQR, 50-70 years old). Approximately 56% (67/119) of the patients were male. Most of the patients had at least one comorbidity, with hypertension [70.6% (84/119)] and diabetes mellitus [44.5% (53/119)] were the most frequent ones. The most common presenting symptoms on initial diagnosis were shortness of breath [60.5% (72/119)], fever [37.0% (44/119)], cough (26.1% [31/119]). There were 77.3% (92/119) patients meeting criteria for severe disease and 59.5% (71/119) admitted to ICU.

**Table 1:** Comparison of baseline characteristics and outcome between COVID-19 patients with elevated and normal fibrinogen.

Variable	Total (n=119)	Fibrinogen (≥489.0mg/dl, n=80)	Fibrinogen (<489.0mg/dl, n=39)	*P* Value
Age (IQR), y	62.0 (50.0-70.0)	62.0 (53.3-70.0)	61.0 (42.0-71.0)	0.301^a^
Male, n (%)	67 (56.3)	47 (58.8)	20 (51.3)	0.555
Race				
African American, n (%)	73 (61.3)	54 (67.5)	19 (48.7)	0.07
White American, n (%)	38 (31.9)	20 (25.0)	18 (46.2)	0.035
Others, n (%)	5 (4.2)	3 (3.8)	2 (5.1)	0.662
Unknown, n (%)	2 (1.7)	2 (2.5)	0 (0)	1
**Comorbidities**				
Hypertension, n (%)	84 (70.6)	61 (76.3)	23 (59.0)	0.058
Diabetes mellitus, n (%)	53 (44.5)	42 (52.5)	11 (28.2)	**0.018**
Coronary artery disease, n (%)	12 (10.1)	7 (8.8)	5 (12.8)	0.525
Chronic renal failure, n (%)	11 (9.2)	7 (8.8)	4 (10.3)	0.749
Chronic obstructive pulmonary disease, n (%)	14 (11.8)	8 (10.0)	6 (15.4)	0.383
History of malignancy, n (%)	18 (15.1)	14 (17.5)	4 (10.3)	0.416
**Symptoms**				
Shortness of breath, n (%)	72 (60.5)	54 (67.5)	18 (48.7)	**0.03**
Fever, n (%)	44 (37.0)	32 (40.0)	12 (30.8)	0.419
Cough, n (%)	31 (26.1)	23 (28.8)	8 (20.5)	0.381
Fatigue, n (%)	18 (15.2)	15 (18.8)	3 (7.7)	0.172
Nausea, n (%)	4 (3.3)	2 (2.5)	2 (5.1)	0.596
Diarrhea, n (%)	9 (7.6)	8 (10.0)	1 (2.6)	0.268
Body ache, n (%)	4 (3.4)	2 (2.5)	2 (5.1)	0.596
Chest pain, n (%)	6 (5.0)	5 (6.3)	1 (2.6)	0.662
Lack of appetite, n (%)	6 (5.0)	6 (7.5)	0 (0)	0.176
**Vital signs**				
Temperature (°C)	37.2 (36.6-38.1)	37.2 (36.7-38.3)	36.9 (36.6-38.0)	0.206^a^
Respiratory rate (breaths/min)	20.0 (17.0-27.0)	20.0 (18.0-28.0)	18.0 (16.0-25.5)	0.067^a^
Blood pressure (mmHg)				
Systolic	128.2 ± 24.7	129.2 ± 25.4	126.1 ± 23.4	0.549^b^
Diastolic	73.2 ± 15.0	72.4 ± 14.4	75.0 ± 16.2	0.419^b^
Heart rate (beats/min)	93 (80-107)	92.0 (80.0-103.0)	95.5 (77.0-110.3)	0.347^a^
Oxygen saturation (%)	96 (93-98.5)	95.0 (90.0-98.0)	97.5 (94.8-99.0)	**0.003^a^**
**Oxygen treatment**				
Room air, n (%)	22 (18.5)	10 (12.5)	12 (30.8)	0.023
Nasal cannula, n (%)	31 (26.1)	19 (23.8)	12 (30.8)	0.505
Ventilation, n (%)	66 (55.5)	51 (63.8)	15 (38.5)	**0.011**
ICU admission	71 (59.7)	55 (68.8)	16 (41.0)	**0.005**
Severe disease, n (%)	92 (77.3)	67 (83.8)	25 (64.1)	**0.021**
Outcomes				
Discharge alive, n (%)	78 (65.5)	50 (62.6)	28 (71.8)	0.412
Death, n (%)	27 (22.7)	20 (25.0)	7 (17.9)	0.344
Still hospitalized, n (%)	14 (11.8)	10 (12.5)	4 (10.3)	1

ICU admission: intensive care unit admission. ^a^Mann-Whitney U test; ^b^Student’s t-test; all other analyses were performed using Chi square test. The bold values indicate P values < 0.05.

Patients’ laboratory findings on initial diagnosis of COVID-19 are shown in **Table 2**. CBC and differential showed that patients had decreases lymphocyte counts [0.9 (0.6-1.2) ×10^9^/L]. Coagulopathy was developed in patients with prolonged prothrombin time (PT) [14.7 (13.5-16.1) sec], elevated fibrinogen (613.0 ± 223.0 mg/dL) and D-dimer (1053.0 [583.0-2382.0] ng/mL). Patients also showed impaired liver function and renal function with elevated aspartate aminotransferase (AST) [41.0 (29.0-68.0) U/L], lactase dehydrogenase (LDH) [391.5 (292.3-610.3) U/L], blood urea nitrogen (BUN) [23.0 (12.5-31.5) mg/dL], and creatinine [1.4 (0.8-2.2) mg/dL]. Elevated glucose [138.0 (107.3-192.8) mg/dL] was also seen in our study. Cardiac involvement was demonstrated with increased B-type natriuretic peptide (BNP) [122.0(51.0-303.0) pg/mL] and high sensitivity troponin-I (hsTnI) [22.0 (9.0-65.5) ng/L]. While the negative acute phase reactant albumin was decreased (3.1 ± 0.7 mg/dL), the positive acute phase reactants showed increased level with increased C-reactive protein (CRP) (188.3 ± 110.5 mg/L), erythrocyte sedimentation rate (ESR) (55.7 ± 32.0 mm/h), ferritin [454.0 (140.0-1620.0) ng/mL], and procalcitonin (PCT) [0.5 (0.2-1.8) ng/mL].

**Table 2:** Comparison of laboratory parameters between COVID-19 patients with elevated and normal fibrinogen.

Laboratory parameters	Total (n=119)	Fibrinogen (≥489.0mg/dL, n=80)	Fibrinogen (<489.0mg/dL, n=39)	*P* Value
White blood cell (4.0-11.0×10^9^/L)	7.7 (5.5-11.1)	8.4 (6.1-12.2)	7.4 (3.8-9.3)	0.314
Neutrophil (1.7-7.0×10^9^/L)	6.2 (4.1-9.6)	7.0 (4.9-10.7)	5.7 (2.5-7.4)	0.134
Lymphocyte (0.9-2.9×10^9^/L)	0.9 (0.6-1.2)	0.8 (0.6-1.0)	1.0 (0.5-1.1)	**0.048**
Neutrophil/Lymphocyte	6.7 (4.2-11.8)	7.3 (5.7-14.8)	5.6 (3.1-8.8)	**0.041**
Monocyte (0.3-0.9×10^9^/L)	0.5 (0.3-0.7)	0.4 (0.3-0.6)	0.5 (0.3-0.7)	0.919
Hemoglobin (13.5-17.0g/dL,M, 11.3-15.2, F)	11.5 (9.9-13.5)	11.9 (8.9-14.0)	10.3 (9.5-11.6)	0.18
Platelet count (150.0-400.0×10^9^/L)	206.4 (156.7-293.8)	250.2 (140.3-318.9)	197.9 (109.1-303.3)	0.16
PT (12.0-14.5 sec)	14.7 (13.5-16.1)	14.5 (13.3-16.5)	14.9 (14.2-16.4)	0.221
aPTT (25.0-35.0 sec)	34.0 (30.0-39.0)	35.0 (30.0-40.0)	37.0 (33.8-40.0)	0.528
Fibrinogen (220.0-498.0 mg/dL)	613.0 ± 223.0	728.8 ± 167.6	351.0 ± 116.6	**<0.001^a^**
D-dimer (0.0-240.0 ng/mL)	1053.0 (583.0-2382.0)	1213.0 (769.0-3741.0)	610.0 (224.5-1867.3)	0.107
ALT(7.0-52.0 U/L)	27.0 (14.5-48.5)	32.0 (22.0-61.0)	25.0 (13.8-132.8)	0.57
AST (12.0-39.0 U/L)	41.0 (29.0-68.0)	51.0 (39.0-88.0)	47.0 (28.5-312.0)	0.482
LDH (120.0-240.0 U/L)	391.5 (292.3-610.3)	458.0 (372.0-690.0)	270.5 (185.0-1009.5)	**0.001**
Total bilirubin (0.3-1.4 mg/dL)	0.6 (0.4-0.8)	0.5 (0.4-0.7)	0.6 (0.5-1.0)	0.17
Direct bilirubin (0.0-0.3 mg/dL)	0.2 (0.1-0.3)	0.2 (0.1-0.3)	0.2 (0.2-0.4)	0.84
Indirect bilirubin (0.2-0.8 mg/dL)	0.4 (0.3-0.5)	0.3 (0.3-0.5)	0.4 (0.3-0.4)	0.19
BUN (5.0-22.0 mg/dL)	23.0 (12.5-31.5)	23.0 (11.0-32.0)	21.5 (18.3-35.8)	0.446
Creatinine (0.7-1.3 mg/dL,M, 0.4-1.2, F)	1.4 (0.8-2.2)	1.3 (0.7-2.2)	1.6 (0.8-3.7)	0.331
Glucose (70.0-100.0 mg/dL)	138.0 (107.3-192.8)	127.0 (111.0-201.0)	126.5 (90.0-164.5)	0.065
BNP (0.0-100.0 pg/mL)	122.0 (51.0-303.0)	105.0 (47.0-178.0)	138.0 (35.0-8909.8)	0.917
High sensitivity Troponin-I (3.0-20.0 ng/L)	22.0 (9.0-65.5)	26.0 (9.0-72.0)	45.0 (10.5-147.5)	0.618
Albumin (3.7-5.5 mg/dL)	3.1 ± 0.7	3.1 ± 0.7	3.2 ± 0.7	0.185^a^
C-reactive protein (0.0-10.9 mg/L)	188.3 ± 110.5	199.3 ± 113.2	113.2 ± 85.8	**<0.001^a^**
ESR (0.0-20.0 mm/h)	55.7 ± 32.0	65.6 ± 30.6	45.3 ± 28.4	**0.003^a^**
Ferritin (11.0-306.8 ng/mL)	454.0 (140.0-1620.0)	697.0 (116.5-1936.3)	676.0 (152.0-1468.0)	**0.016**
Procalcitonin (0.00-0.07 ng/mL)	0.5 (0.2-1.8)	1.1 (0.1-2.0)	0.4 (0.2-1.2)	**0.016**

PT, prothrombin time; aPTT, activated partial thromboplastin time; ALT, alanine transaminase; AST, aspartate aminotransferase; LDH, lactase dehydrogenase; BUN, blood urea nitrogen; BNP, B-type natriuretic peptide; ESR, Erythrocyte sedimentation rate; F, female; M, male; ^a^Student’s-t test, all other analyses were performed using Mann-Whitney U test. The bold values indicate P values < 0.05.

### Fibrinogen-Associated Factors in COVID-19 Patients

**Figure 1:**
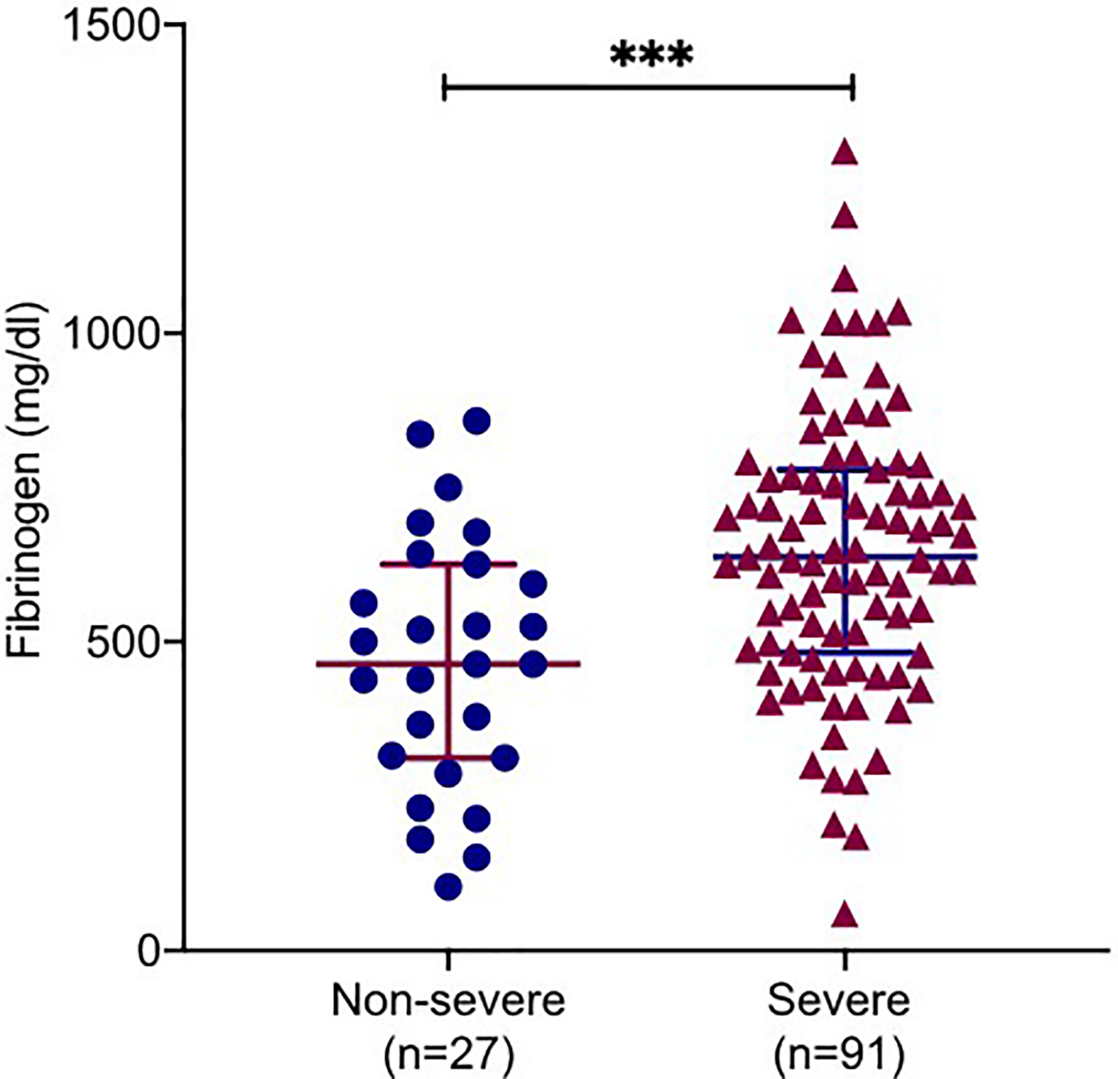
Correlations of fibrinogen levels with clinical severity. Plasma fibrinogen levels in COVID-19 patients with severe and non-severe. All data points are shown and mean ± 95% confidential intervals (CI) are shown by horizontal bars. student *t*-test was performed to determine the statistical significance. Here, *** indicate a *P* value < 0.001.

**Figure 2:**
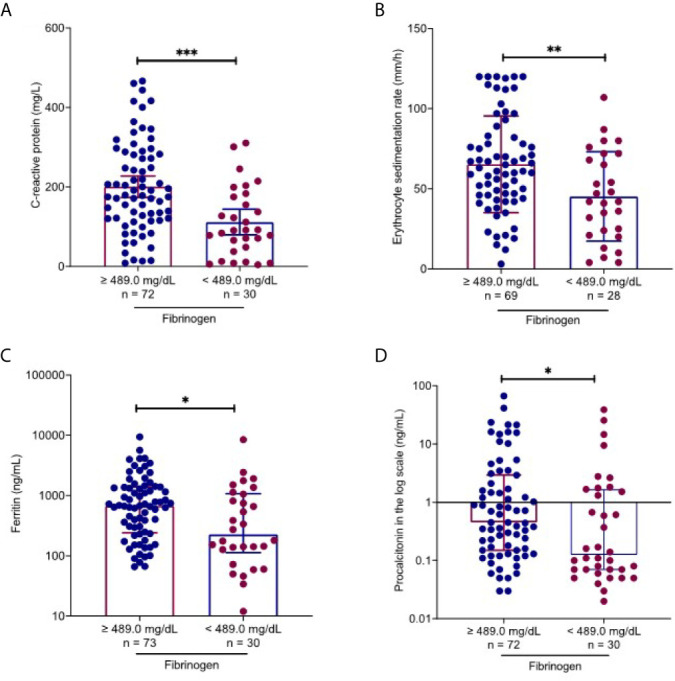
Fibrinogen-associated factors in COVID-19 patients. Using the upper level of the reference laboratory 489.0 mg/dl, inflammatory markers were compared between patients with normal and elevated fibrinogen levels. Patients with elevated fibrinogen levels had higher C-reactive protein **(A)**, erythrocyte sedimentation rate **(B)**, ferritin **(C)** and procalcitonin **(D)** levels than those with normal fibrinogen levels. All data points are shown, and medians ± interquartile range are shown by horizontal bars. Mann-Whitney *U* test was performed to determine the statistical significance. Here, *, ** and *** indicate a *P* value < 0.05, 0.005 and 0.001, respectively.

On admission, 67.2% patients (80/119) had increased fibrinogen (normal range 220.0-489.0 mg/dL), and 32.8% patients (39/119) had fibrinogen levels within the normal range. Fibrinogen levels were significantly associated with disease severity of COVID-19 (**Figure 1**). Patients with severe disease had increased fibrinogen levels than non-severe patients (648.1 mg/dl vs 468.9 mg/dl, *P* = 0.001). To understand the role of fibrinogen in the pathogenesis of COVID-19, the clinical characteristics and laboratory parameters were compared between normal and elevated fibrinogen group (**Tables 1** and **2**). When comparing the clinical characteristics, patients with increased fibrinogen levels were more likely to be white (20 vs 18, P =0.035) and had a higher probability of having a comorbidity of diabetes mellitus (42 vs 11, P =0.018), shortness of breath (54 vs 18, P =0.03), lower oxygen saturation (95.0 vs 97.5, P 0.003), mechanical ventilation (51 vs 15, P=0.011), ICU admission (55 vs 16, P=0.005), and severe disease (67 vs 25, P=0.021) (**Table 1**). When comparing the laboratory parameters, patients with increased fibrinogen levels showed lower lymphocyte count (0.8 [0.6-1.0] vs 1.0[0.5-1.1], P=0.048), higher neutrophil/lymphocyte ratio (7.3 [5.7-14.8] vs 5.6 [3.1-8.8], P=0.041), higher LDH (458.0 [372.0-690.0] vs 270.5 [185.0-1009.5], P=0.001), higher CRP (199.3 ± 113.2 vs 113.2 ± 85.8, P<0.001), higher ESR (65.6 ± 30.6 vs 45.3 ± 28.4, P=0.003), higher ferritin (697.0 [116.5-1936.3] vs 676.0 [152.0-1468.0], P=0.016), and higher PCT (1.1 [0.1-2.0] vs 0.4 [0.2-1.2], P=0.016). Comparison of the inflammatory markers between patients with normal and elevated fibrinogen levels were further demonstrated in **Figure 2**. However, comparison of other coagulation markers and cardiac markers between patients with normal and elevated fibrinogen levels did not show no significant difference.

### Elevated Fibrinogen Levels to Predict Disease Severity and ICU Admission

**Figure 3:**
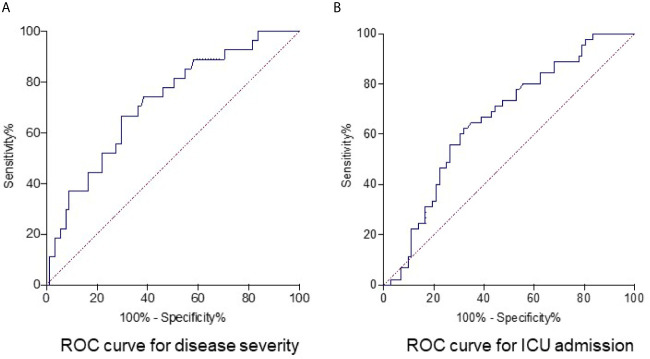
Receiver operating characteristics curve for fibrinogen as parameter for predicting disease severity and ICU admission in COVID-19 patients. The optimum cutoff point of fibrinogen to predict severe disease in COVID-19 was 528.0 mg/dl, with 66.7% for sensitivity and 70.3% for specificity. Area under receiver operator characteristic curve (AUC) was 0.72 and *P* value was 0.0006 **(A)**. The optimum cutoff point of fibrinogen to predict ICU admission of COVID-19 patients was 571.0 mg/dl, with a sensitivity and specificity of 62.2% and 68.1%. The AUC was 0.66 and *P* value was 0.0037 **(B)**.

Next, the ROC analysis was performed to evaluate the role of fibrinogen in prediction of disease severity. The results showed that fibrinogen at 528.0 mg/dl on admission as the optimal cutoff level to discriminate severe from non-severe patients (AUC, 0.72; standard error [SE], 0.05; 95% confidence interval [CI], 0.61-0.83; *P* = 0.0006), with a sensitivity and specificity of 66.7% and 70.3%, respectively (**Figure 3A**). Seventy seven percent (92/119) patients had a fibrinogen > 528.0 mg/dl in this study cohort. The optimal cutoff for fibrinogen to predict ICU admission in our cohort was 571.0 mg/dl (AUC, 0.66; SE, 0.05; 95% CI, 0.58-0.76; *P* = 0.0037), with a sensitivity of 62.2% and specificity of 68.1% (**Figure 3B**).

## Discussion

In this retrospective study, we assessed the distribution of fibrinogen in a cohort of COVID-19 patients. It is worth noting that fibrinogen levels are elevated in COVID-19 patients upon admission, especially in those with severe disease. Fibrinogen levels on admission were associated with elevated inflammatory markers, and elevated fibrinogen levels may be used as an early biomarker to predict disease severity and ICU admission.

COVID-19 is a systemic infection with significant impact on the coagulation system that often manifests in thrombotic complications and coagulopathy (Levi et al., 2020). Previous data have showed that fibrinogen levels are elevated among patients with COVID-19 (Toh and Hoots, 2007; Han et al., 2020) and decreased at later stage of the disease when disseminated intravascular coagulation happens (Tang et al., 2020). However, there is no consensus on the role of fibrinogen in predicting outcomes in COVID-19 patients regarding disease severity, ICU admission and mortality (Gao et al., 2020; Liu et al., 2020; Tang et al., 2020). Tang et al. have reported that fibrinogen levels are elevated in both survived and non-survived group, but the difference between two groups was not statistically significant (Tang et al., 2020). However, studies by other groups reported an association between elevated fibrinogen levels upon admission and poor outcome (Gao et al., 2020; Liu et al., 2020). In this study, we report that elevations in fibrinogen upon admission were significantly associated with disease severity and ICU admission in COVID-19 patients. Consistently, we found that LDH, a biomarker for severe disease or multiple organ failure was significantly associated with the fibrinogen level in our study.

Infection with bacterial, viral, or fungal pathogens initiates the systemic inflammation response. Significant inflammation is present in patients with 2019-nCoV/SARS-CoV-2 infection, based on increased IL-6, CRP and ESR levels upon admission (Chen et al., 2020a). In our study, the decreased negative acute phase reactant albumin and increased positive acute phase reactant CRP, ferritin, ESR and PCT indicated a systemic inflammation response in the patients. In COVID-19 patients with acute respiratory distress syndrome (ARDS), fibrinogen levels were reported to be associated with elevated interleukin-6 values (Ranucci et al., 2020). In the present study, we found that acute inflammatory markers, including CRP, ferritin, ESR and PCT were all significantly associated with elevated fibrinogen. RK Mahat et al. performed a meta-analysis of inflammatory markers in COVID-19, and they found that the inflammatory markers CRP, ferritin, ESR and PCT were associated with disease severity. However, fibrinogen was not analyzed in their study (Mahat et al., 2021).

Myocardial injury has been recognized as a common complication in COVID-19 patients (Boukhris et al., 2020). Myocardial injury is defined as the presence of at least one cardiac troponin value above the 99th percentile upper reference limit (URL) according to the Fourth Universal Definition of Myocardial Infarction (Thygesen et al., 2018). Consistent with many other reports (Lombardi et al., 2020; Garcia De Guadiana-Romualdo et al., 2021; Majure et al., 2021), elevated hsTnI was detected in the COVID-19 patients in our study. The mechanisms for myocardial injury in COVID-19 has not been elucidated. Several possible mechanisms have been proposed such as direct virus attack, inflammation-related injury, coronary plaque rupture, and microvascular thrombosis (Babapoor-Farrokhran et al., 2020; Imazio et al., 2020; Tajbakhsh et al., 2021). Fibrinogen may increase cardiovascular risk due to adverse effects of fibrinogen on plasma viscosity, coagulation, platelet activity, inflammation and atherogenesis (Kakafika et al., 2007). However, the direct myocardial injury biomarker, hsTnI, did not show significant association with fibrinogen level in our study (P=0.087).

The study has several limitations. First, this study was a single-center, retrospective study which needs to be further validated in a prospective study. Second, a proportion of asymptomatic, mild or moderate patients who did not have fibrinogen levels evaluated on admission were excluded from final analysis, which may underestimate the differences between groups. Third, dynamic changes of fibrinogen during the clinical course might also provide more information in the outcome of the patients.

In conclusion, our findings indicated that fibrinogen level on admission were associated with elevated inflammatory markers in COVID-19 patients, and elevated fibrinogen levels may predict severe disease and ICU admission. Monitoring fibrinogen level could be helpful in management of COVID-19 patients.

## Data Availability Statement

The original contributions presented in the study are included in the article/supplementary material. Further inquiries can be directed to the corresponding author.

## Ethics Statement

The studies involving human participants were reviewed and approved by University of Alabama at Birmingham Institutional Review Board. Written informed consent for participation was not required for this study in accordance with the national legislation and the institutional requirements.

## Author Contributions

JS and LC designed the study, collected the data and drafted the manuscript. DN and SG revised the manuscript. All authors contributed to the article and approved the submitted version.

## Conflict of Interest

The authors declare that the research was conducted in the absence of any commercial or financial relationships that could be construed as a potential conflict of interest.

## Publisher’s Note

All claims expressed in this article are solely those of the authors and do not necessarily represent those of their affiliated organizations, or those of the publisher, the editors and the reviewers. Any product that may be evaluated in this article, or claim that may be made by its manufacturer, is not guaranteed or endorsed by the publisher.
